# Improving Postharvest Quality of ‘Tango’ Mandarin Using a Preharvest Sonicated Nanoemulsion-Based Delivery System of Methyl Jasmonate and 1-Naphthaleneacetic Acid

**DOI:** 10.3390/foods15091612

**Published:** 2026-05-06

**Authors:** Muhammad Nadeem, KeAndre Leaks, Julia Sage Adamson Felix, Tahir Mahmood Qureshi, Ahmed Abdullah, Zafar Iqbal, Muhammad A. Shahid

**Affiliations:** 1 Horticultural Science Department, North Florida Research and Education Center, Institute of Food and Agricultural Sciences, University of Florida, Quincy, FL 32351, USA; nadeem.muhammad@ufl.edu (M.N.); leaks.k@ufl.edu (K.L.); js.adamsonfelix@ufl.edu (J.S.A.F.); ahmedabdullah@ufl.edu (A.A.); 2 Institute of Food Science and Nutrition, University of Sargodha, Sargodha 40100, Pakistan; 3 Department of Food Sciences, Cholistan University of Veterinary and Animal Sciences, Bahawalpur 63100, Pakistan; tahirmahmood@cuvas.edu.pk; 4 Central Laboratories, King Faisal University, Al-Ahsa 31982, Saudi Arabia

**Keywords:** nanoemulsion, sonication, antioxidants, degreening, methyl jasmonate, 1-naphthaleneacetic acid, fruit quality

## Abstract

The use of nanotechnology-based delivery systems along with growth regulators of plants is a promising approach to increase the quality of fruits postharvest. This experiment was aimed at determining the impact of sonicated nanoemulsions containing methyl jasmonate (MeJA) and 1-naphthaleneacetic acid (NAA) on the quality of postharvest Tango mandarin. The findings showed the samples treated improved fruit weight, diameter, firmness and juice percentage significantly compared to the control sample. TMJ4 (10.0 µM L^−1^) was the most effective treatment at preserving the fruit firmness, whereas TMJ3 (7.5 µM L^−1^) was the best treatment at improving the internal quality characteristics, such as total soluble solids, sugar–acid ratio, phenolics, flavonoids, flavonols, and antioxidant activity. Reduction in titratable acidity and a subsequent increase in sugar-to-acid ratio indicated a better maturation behavior and flavor profile of the fruit. The augmented retention of bioactive compounds implies the stimulation of secondary metabolite pathways and stress tolerance. The results suggest that nanoemulsion delivery of methyl jasmonate and NAA by sonication is an efficient method of enhancing the quality of the postharvest and prolonging the shelf life of Tango mandarin.

## 1. Introduction

Tango mandarin (*Citrus reticulata* Blanco) is a seedless triploid cultivar derived from irradiation-induced mutation of *W. Murcott Afourer*. It has become a preferred choice in commercial citrus orchards for its superior consumer traits such as rich flavor, ease of peeling and absence of seeds even under open-pollinated conditions. Its mid- to late-season harvest window (January to March) offers an opportunity to extend market availability and avoid competition with early-season cultivars like Satsuma and Clementine. The fruit’s moderate canopy vigor, pollen sterility, and relatively uniform fruit size further support its cultivation in subtropical and Mediterranean climates [1].

However, the commercial value of Tango mandarin is quite high, but uneven peel coloration and poor postharvest characteristics are still serious issues, especially in subtropical climatic conditions. Most traditional postharvest interventions, such as ethylene degreening, have inconsistent outcomes due to limited cultivar response to the treatment [2]. Moreover, environmental factors may also influence pigment biosynthesis. These constraints underscore the necessity to adopt other measures having the potential to control physiological and biochemical processes that are linked to the process of fruit ripening and quality improvement [3].

Methyl jasmonate (MeJA) and 1-naphthaleneacetic acid (NAA) have been widely reported as plant growth regulators having a significant impact on fruit development and ripening via regulating hormonal signaling pathways [4]. Methyl jasmonate has an essential role in triggering the breakdown of chlorophyll, carotenoid production and antioxidant defense response, thus leading to enhanced coloration and stress tolerance [5]. Equally, NAA (as an artificial auxin) can control cell expansion, assimilate partitioning and metabolic processes linked to the fruit development and maturation [6]. The utility of these compounds is, however, usually restricted by their low stability, quick breakdown and ineffective penetration into plant tissues when used in traditional ways.

The new developments in nanotechnology have brought the use of nanoemulsion-based delivery systems as one of the ways of overcoming these limitations. Small droplet length leads to a large surface area, increasing the bioavailability, stability, and solubility of hydrophobic compounds in nanoemulsions [7]. Sonication-aided nanoemulsification not only enhances the stability of dispersion but also causes rapid uptake and sustained encapsulation of bioactive compounds in plant tissues [8].

Nevertheless, few investigations have been done to examine how sonicated nanoemulsions can be used synergistically with plant growth regulators to enhance the quality of Tango mandarin after harvest [9]. Thus, the current experiment aimed to determine the efficacy of methyl jasmonate and 1-naphthaleneacetic acid added to sonicated nanoemulsions in improving the physical, biochemical, and antioxidant properties of fruit, which is a new and sustainable method to manage its quality during postharvest [10].

The recent developments in nanotechnology have opened new horizons for increasing the efficiency of plant growth regulators (PGRs). Colloidal dispersions of oil and water stabilized with surfactants, called emulsions, have raised interest because of having large surface areas, better solubility of hydrophobic materials, and greater permeability in plant cells [11]. Sonicated nanoemulsions have smaller droplets (20–200 nm) with a high degree of stability that can be used to deliver bioactive compounds in agricultural systems in much-controlled and targeted ways [12]. In this way, sonicated nanoemulsions will not only enhance the bioavailability of encapsulated molecules but also reduce the amount of dosage, thus resulting in an environmentally sustainable form of crop management.

Another application of nanoparticle-based delivery systems in fruit physiology is the enhanced intake and efficacy of phytohormones, antioxidants, and essential oils, which leads to better postharvest quality and stress tolerance [13]. In order to increase the physiological effect of nanoemulsions by sonication, it is possible to integrate methyl jasmonate (MeJA) and 1-naphthaleneacetic acid (NAA), which could increase the depth of permeation of the nanoemulsions in the tissues of fruits and extend their activity. This may have the advantages over conventional sprays, which may exhibit uneven application, photodegradation, and metabolize quickly, resulting in limited efficacy.

Nano-delivery systems combined with phytohormones are a potential strategy with great possibilities of improving bioavailability, stability and physiological efficacy, but their use in citrus postharvest management is not fully studied [14]. Considering these developments, the use of nanoemulsion as a base could be an eco-friendly solution to the long-term issue of delayed and uneven peel color in Tango mandarin grown in subtropical regions. Sonicated nanoemulsions have the potential to prevent external discoloration and improve internal quality by balancing the hormonal regulation of MeJA and NAA. Thus, this experiment was designed to identify the effects of MeJA and NAA integrated in sonicated nanoemulsion on the development of peel colors of Tango fruit and other fruit quality parameters.

## 2. Materials and Methods

### 2.1. Materials

Citrus trees of the Tango variety were selected for the experiment from the citrus variety evaluation block at the North Florida Research and Education Center, University of Florida, Quincy, Florida. Citrus peel oil was procured from the manufacturer Nature’s Oil (115 Lena Drive, Aurora, OH, USA). Sodium hydroxide, sodium carbonate, sodium phosphate (Na_2_HPO_4_·12H_2_O), phenolphthalein, carboxymethyl cellulose, glycerol, Folin–Ciocalteu reagent, ethanol, DPPH, sulphuric acid, ammonium molybdate, catechin, gallic acid, sodium nitrite, ascorbic acid, quercetin, Polysorbate, Methyl jasmonate, 1-naphthaleneacetic acid (NAA) and aluminum chloride were purchased from Sigma Aldrich, St. Louis, MO, USA. All chemicals were of analytical grade.

### 2.2. Methods

#### 2.2.1. Development of Sonicated Emulsion

Sonicated emulsion was prepared by emulsifying 500 mg carboxymethyl cellulose (CMC) in 100 mL distilled water along with 750 µL glycerol, 2000 µL Tween-80 and 500 µL orange peel oil. The emulsification and homogenization of the aforementioned ingredients were carried out in a blender (Waring MX1050XTS Xtreme, McConnellsburg, PA, USA) at 30,000 rpm for 5 min. The resulting emulsion was then ultrasonicated using a 15 mm probe at a frequency of 20 kHz, amplitude of 70%, and power of 950 W and pulse mode (5 s on, 5 s off) at 20 ± 1 °C for 10 min [15].

The process consists of a high-speed homogenization step to form an emulsion and a further ultrasonication step to decrease the droplet size. Reproducibility is obtained by the combination of correct sonication parameters and controlled processing situations, and the protocol is consistent with typical protocols applied in the nanoemulsion preparation. Ultrasonication facilitates a decrease in droplet diameter and an increase in dispersion stability, and enhances bioavailability and delivery of the plant growth regulators [16]. The above-mentioned factors lead to improved penetration, release, and stimulation of the physiological activity of the treatments being applied.

An emulsification method involving sonication was used to formulate the dispersion, which is commonly known to generate nanoscale dispersion, but no direct physicochemical characterization (e.g., droplet size, polydispersity index, or zeta potential) was done in the current study. Hence, the word nanoemulsion used represents the preparation procedure but does not establish nanoscale characteristics [17].

#### 2.2.2. Treatment Plan

The study followed a completely randomized design (CRD) with 2 types of plant biostimulants with variable levels, and each level was replicated three times.

#### 2.2.3. Experimental Design

Citrus trees were divided into different treatment groups, and experiments started 2 months prior to harvesting. Further, the experiments were divided into the following groups:

Control group: Citrus fruits on trees were sprayed with distilled water.

Methyl jasmonate treatment: Citrus fruits on trees were sprayed with MJ incorporated in a sonicated nanoemulsion with varying levels (Table 1).

1-Naphthaleneacetic acid (NAA) treatment: Citrus fruits on trees were sprayed with 1-NAA incorporated in a sonicated nanoemulsion according to the treatment plan (Table 1).

The experiments were carried out through a completely randomized design (CRD) where the environmental conditions were homogeneous to guarantee that the experimental units were similar. Eight treatments (one of them has a control) with four levels of methyl jasmonate (MeJA) and three levels of 1-naphthaleneacetic acid (NAA) were used. Every treatment was repeated three times.

Each treatment took experimental units (citrus trees/fruits) to have independent observations as well as to reduce bias. The number of replicates represents a set of an equal number of fruits under equal conditions of treatment. Treatments were as follows: control (distilled water), TMJ1 (2.5 µM L^−1^ MeJA), TMJ2 (5.0 µM L^−1^ MeJA), TMJ3 (7.5 µM L^−1^ MeJA), TMJ4 (10.0 µM L^−1^ MeJA), TNA1 (300 µM NAA), TNA2 (400 µM NAA), and TNA3 (500 µM NAA).

All the treatments were performed in the same agronomic and environmental conditions to make them consistent. The CRD was deemed to be relevant because the conditions of the experimental field were relatively unified. The design enabled the objective estimation of the effects of treatments, and the statistical analysis was based on analysis of variance (ANOVA).

Each replicate comprised a homogeneous number of fruits that were picked in randomly chosen trees allotted to a particular treatment. Each replicate contained a fixed number of fruits (n = 20) to have a representative measure of the parameters of quality. These fruits were viewed as subsamples in the individual experimental units, and statistical analysis was done on the average values of these fruits. The sampling was done at certain intervals in terms of the fruit development (0, 30 and 60 days) in order to study the time-related changes in the response based on the treatments.

#### 2.2.4. Physical Quality Parameters of Fruits

Measurement of all the physical quality parameters was done on standardized and calibrated instruments to achieve reproducibility and accuracy. A digital scale Model H-1651 (Made in China, distributed by ULINE, Pleasant Prairie, WI, USA) was used to determine the fruit weight and peel weight. The weight of each entire fruit and its peel was measured and expressed in grams (g). The diameter of the fruit was taken using a digital vernier caliper at the equatorial area (mid point), and the polar area and the mean of the fruit were calculated as the average diameter of the fruit. All samples were analyzed using the same protocol in order to maintain consistency. The fruit firmness (kg cm^−2^) was determined by using a penetrometer (model FT-327, Facchini srl, Alfonsine, Italy) having a cylindrical probe (8 mm diameter) on the two opposite sides of each fruit to consider the peel heterogeneity, and the average of both readings was expressed as firmness [18]. After measuring fruit firmness, citrus fruits were subjected to juice extraction using a citrus squeezer (MJ-M176P from Panasonic Berhad, Shah Alam, Malaysia), and the volume of juice obtained was measured (mL) in a glass cylinder. The percentage of juice was determined using the relationship between the volume of juice and the fruit weight. The quality parameters associated with juice were measured. The TSS of juice was measured by using a refractometer (Atago, Tokyo, Japan) that was calibrated. Some of the extracted juice was placed on the surface of the prism, and the TSS was estimated. Titratable acidity (TA) was determined by following the standard titration protocol and was expressed as anhydrous citric acid (%). Each juice sample containing phenolphthalein indicator was titrated against 0.1 N NaOH solution until the pink endpoint (pH 8.2), and the results were calculated using the formula [19]. TSS/acidity was determined by dividing the TSS by TA.

(1) $$\mathrm{Acidity}\;(\%)=\frac{Titer\;value\;(mL)\times 0.0064}{10\times 100}$$

#### 2.2.5. Estimation of Phytochemical Parameters

##### Determination of Total Phenolic Content (TPC)

The Folin–Ciocalteu method was used to determine the total phenolic content (TPC). In the first step, 1000 µL of Folin–Ciocalteu reagent (10%) was combined with 500 µL of the juice sample. After 6 min, 2000 µL sodium carbonate solution (20%) was added, and kept for 1 h at 30 °C in the dark. Absorbance was determined at 760 nm by a microplate reader, and the outcomes were given in the form of mg gallic acid equivalents (GAEs) per 100 mL [20].

##### Determination of Total Flavonoids

The aluminum chloride colorimetric method [15] was used to determine the total flavonoid content (TFC). The mixture containing 250 µL juice sample, 1250 µL distilled water, and 75 µL sodium nitrite was prepared. Aluminum chloride (150 µL) and sodium hydroxide (500 µL) 1 M were added after 6 min. The 10 mL of distilled water was added to the volume, and the absorbance was recorded at 510 nm. The results were given in terms of mg catechin equivalents (CE)/100 mL.

##### Determination of Total Flavanols

The total flavonol content (TFl) was calculated by adding 1 mL of the sample of juice into 1 mL of aluminum chloride (2%) and 3 mL of sodium acetate (5%), and incubating them in the dark at 23 °C for 30 min. Absorbance was measured at 430 nm, and the results were presented in mg quercetin equivalent (QE)/100 mL [15].

##### Determination of Total Antioxidant Activity

The phosphomolybdenum complex method [21] was used to measure total antioxidant activity (TAA). A 0.4 mL sample of juice was mixed with 4.0 mL of reagent solution, the tubes were incubated at 90 °C for 95 min, and the absorbance was determined at 695 nm. The results were reported as mg ascorbic acid equivalent (AAE)/100 mL.

##### Determination of DPPH Radical Scavenging Activity

DPPH radical scavenging activity was measured by following the procedure of [22]. In short, 2 mL of juice was reacted with 2 mL of 0.2 mM DPPH solution. The resultant solution was then kept in darkness for half an hour, after which the absorbance was taken using a 96-well microplate reader at 517 nm. DPPH solution without a sample was taken as a control, and radical scavenging activity (RSA) was determined as

(2) $$\mathrm{RSA}\;(\%)=\frac{A_{control}-A_{sample}}{A_{sample}}\times 100$$

A_control_ = absorbance of the control, and A_sample_ = absorbance of the sample. The same procedure was used to analyze ascorbic acid standards, and the results were represented in percent of inhibition.

##### Statistical Analysis

The data obtained based on all measured parameters were analyzed using a completely randomized design of analysis of variance (ANOVA) in the Minitab 16 statistical software. The effects of the treatment and intervals between the samples in terms of time were compared, and the mean value of both samples was compared through the Tukey honest significant difference (HSD) test at the *p* < 0.05 level [23]. Each and every measurement was done thrice, and the results were expressed in the form of mean and standard deviation (SD). Subsampling in each of the replicates provided better accuracy and less variation in the data set.

## 3. Results

### 3.1. Effect of Treatments on Fruit Weight

**Figure 1:**
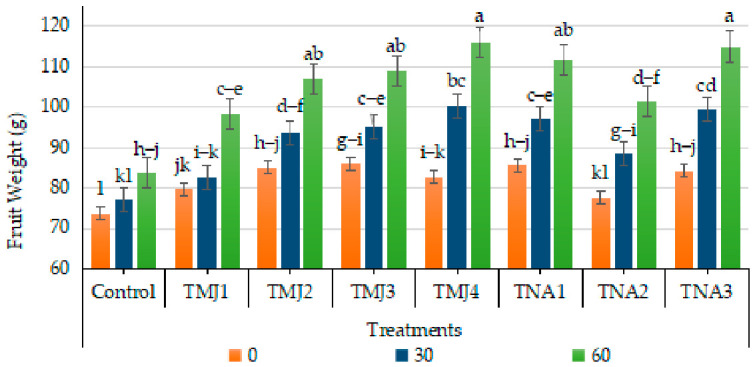
Effect of sonicated nanoemulsion integrated with methyl jasmonate and 1-Naphthalein Acetic Acid on fruit weight (g). The values are presented as mean ± SD. Different letters show a significant difference among treatments at *p* ≤ 0.050. Control: untreated, TMJ1: 2.5 µM L^−1^, TMJ2: 5.0 µM L^−1^, TMJ3: 7.5 µML^−1^, TMJ4: 10.0 µM L^−1^, TNA1: 300 µM, TNA2: 400 µM, and TNA3: 500 µM.

The use of sonicated nanoemulsions containing methyl jasmonate (MeJA) and 1-naphthalene acetic acid (NAA) showed significant improvement in fruit weight (Figure 1). The weight of all the treated fruits was higher than that of the control (up to 13.57%), which suggests improved uptake and bioavailability using nanoemulsions. MeJA treatments TMJ4, TMJ3, TMJ2, and TMJ1 showed the more significant increase in fruit weight (40.32, 26.74, 25.88, and 23.42%, respectively). Fruit weight was also improved by applying 1-NAA treatments. Among the treatments, TNA3 (500 µM 1-naphthalene acetic acid) performed the best, resulting 36.37% more gain in fruit weight as compared to control untreated fruits. The findings affirmed that the sonicated nanoemulsion delivery method improves stability and physiological activity of growth regulators, and aids in fruit growth and yield potential.

### 3.2. Effect of Treatments on Fruit Diameter

**Figure 2:**
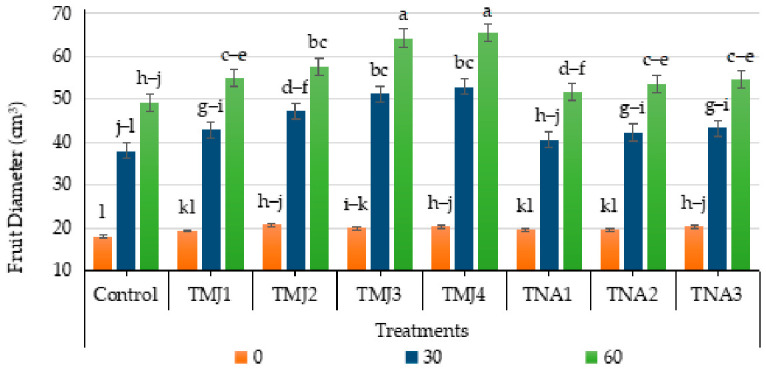
Effect of sonicated nanoemulsion integrated with methyl jasmonate and 1-Naphthalein Acetic Acid on fruit diameter (cm^3^). The values are presented as mean ± SD. Different letters show a significant difference among treatments at *p* ≤ 0.050. Control: untreated, TMJ1: 2.5 µM L^−1^, TMJ2: 5.0 µM L^−1^, TMJ3: 7.5 µM L^−1^, TMJ4: 10.0 µM L^−1^, TNA1: 300 µM, TNA2: 400 µM, and TNA3: 500 µM.

It was observed that sonicated nanoemulsions with plant growth regulators significantly improved radial development of fruits (Figure 2). The results of ANOVA revealed that the effects of both the treatment and days were highly significant (*p* < 0.001) on the fruit diameter, but the interaction between the two factors was nonsignificant. The HSD test revealed that group A (TMJ4) had the highest fruit diameter, which was significantly different from the control (Group D). The value of the CV is low (10.05%), indicating a good experimental precision. Fruit diameter in the control treatment increased to 174.06% after 60 days. Among MeJA treatments, TMJ4 showed the highest increase (223.02%) in fruit diameter, which was 48.96% more than the control treatment. However, 1-naphthalene acetic acid treatments also exhibited a significant effect on fruit diameter as compared to the control, but with a relatively lower level of effectiveness as compared to MeJA treatments. So, methyl jasmonate demonstrated enhanced effectiveness in supporting the increase in fruit dimensional growth, indicating higher efficiency in prompting physiological activities related to fruit development.

### 3.3. Effect of Treatments on Peel Weight

**Figure 3:**
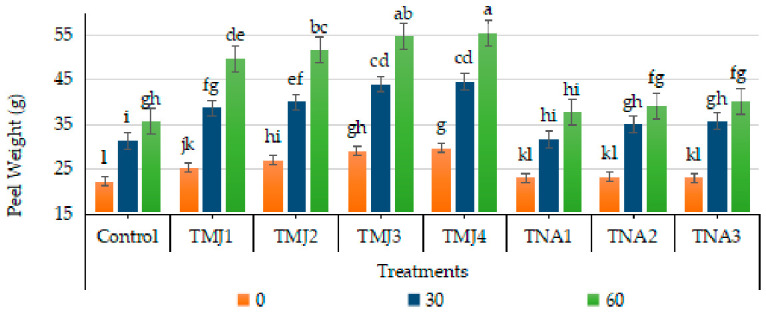
Effect of sonicated nanoemulsion integrated with methyl jasmonate and 1-Naphthalein Acetic Acid on peel weight (g). The values are presented as mean ± SD. Different letters show a significant difference among treatments at *p* ≤ 0.050. Control: untreated, TMJ1: 2.5 µM L^−1^, TMJ2: 5.0 µM L^−1^, TMJ3: 7.5 µM L^−1^, TMJ4: 10.0 µM L^−1^, TNA1: 300 µM, TNA2: 400 µM, and TNA3: 500 µM.

The sonicated nanoemulsions containing methyl jasmonate (MJ) or 1-naphthalene acetic acid (NAA) exhibited a highly significant impact on the peel weight (Figure 3). The treatment, days, and their interaction had a substantial influence on peel weight (*p* < 0.01). HSD test by Tukey cluster TMJ4 and TMJ3 in the highest category (Group A), and it showed better performance than control and NAA treatments. The CV value (7.21) proves good data consistency. The lowest increase in peel weight (75.45%) was recorded in the control treatment after 60 days. The highest increase was observed in TMJ4 (86.48%), which revealed 11.0% more peel weight as compared to the control after 60 days of spray treatment. On the other hand, 1-NAA treatments showed relatively less but significant gains in peel weight. The results demonstrated that the use of methyl jasmonate and 1-NAA may be involved in progressive improvement of fruit weight as well as peel weight by regulating cell expansion.

### 3.4. Effect of Treatments on Fruit Firmness

**Figure 4:**
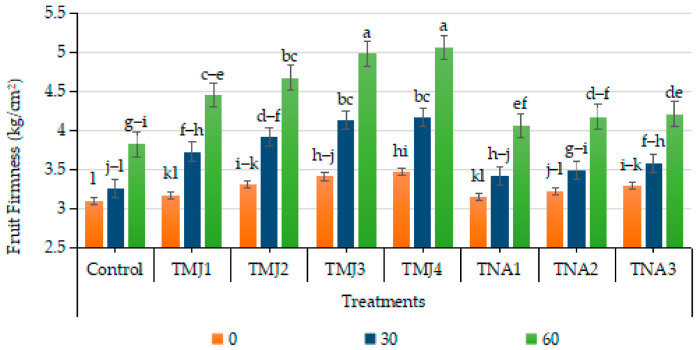
Effect of sonicated nanoemulsion integrated with methyl jasmonate and 1-NaphthaleinAcetic Acid on fruit firmness (kg/cm^2^). The values are presented as mean ± SD. Different letters show a significant difference among treatments at *p* ≤ 0.050. Control: untreated, TMJ1: 2.5 µM L^−1^, TMJ2: 5.0 µM L^−1^, TMJ3: 7.5 µM L^−1^, TMJ4: 10.0 µM L^−1^, TNA1: 300 µM, TNA2: 400 µM, and TNA3: 500 µM.

Sonicated nanoemulsion treatments and days had a significant impact on the fruit firmness (*p* < 0.001), whereas the interaction effect was not significant. Tukey HSD test showed that TMJ4 and TMJ3 were in the top statistical group (A), which indicated a significantly higher firmness than the control (Group E). The CV (5.30) is low, which means that there is high reliability of the data (Figure 4). The control treatment had the least increase in firmness (23.23%), but MeJA treatments had a significant effect on firmness. Among treatments of MeJA, TMJ3 exhibited the highest increase in firmness (46.04%), i.e., 22.81% higher than the control group. Treatments with 1-NAA also increased firmness by about 5.87% as compared to the control. The increased firmness in MeJA and 1-NAA treatments confirmed the efficacy of sonicated emulsion-based delivery in improving fruit firmness, which underscores their possible use in prolonging fruit storage and preserving their quality.

### 3.5. Juice Quality Parameters

#### 3.5.1. Effect of Treatments on Juice Percentage

**Figure 5:**
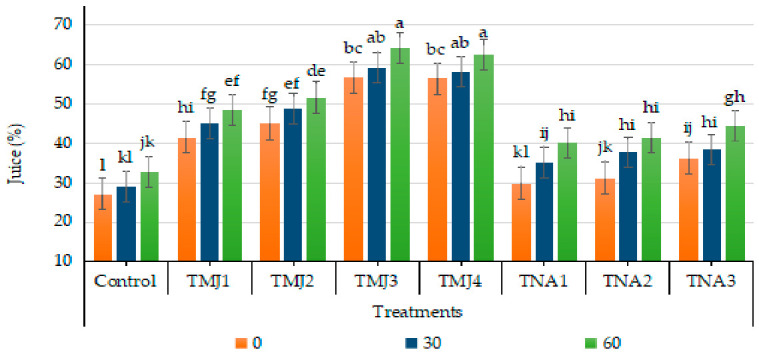
Effect of sonicated nanoemulsion integrated with methyl jasmonate and 1-NaphthaleinAcetic Acid on juice (%). The values are presented as mean ± SD. Different letters show a significant difference among treatments at *p* ≤ 0.050. Control: untreated, TMJ1: 2.5 µM L^−1^, TMJ2: 5.0 µM L^−1^, TMJ3: 7.5 µM L^−1^, TMJ4: 10.0 µM L^−1^, TNA1: 300 µM, TNA2: 400 µM, and TNA3: 500 µM.

Sonicated nanoemulsion integrated with methyl jasmonate and 1-naphthalene acetic acid treatments and days had a significant effect on juice percentage (*p* < 0.001) but no significant interaction effect. The HSD test by Tukey revealed that TMJ3 and TMJ4 were classified in the top group (A), and the treatments performed significantly better compared to other treatments. The CV (3.66) is very low; this indicates great precision in the experiment (Figure 5). Although juice percentage was gradually increased (41.46%) with fruit development under control conditions, the values remained lower than those of the treated samples. The highest juice percentage (62.50%) was recorded in the TMJ4 treatment, showing 21.0% higher juice percentage compared to the control after 60 days of spray treatment. Similarly, 1-naphthalene acetic acid treatments also improved juice percentage compared to the control treatment. Among the 1-NAA-treated samples, TNA3 exhibited the highest juice percentage (44.31%), which was 3.0% higher than the control. The results clearly demonstrated that sonicated nanoemulsion integrated with methyl jasmonate and 1-naphthalene acetic acid significantly enhanced juice percentage and maintained increasing trends during the fruit development stage.

#### 3.5.2. Effect of Treatments on Total Soluble Solids (°Brix)

**Figure 6:**
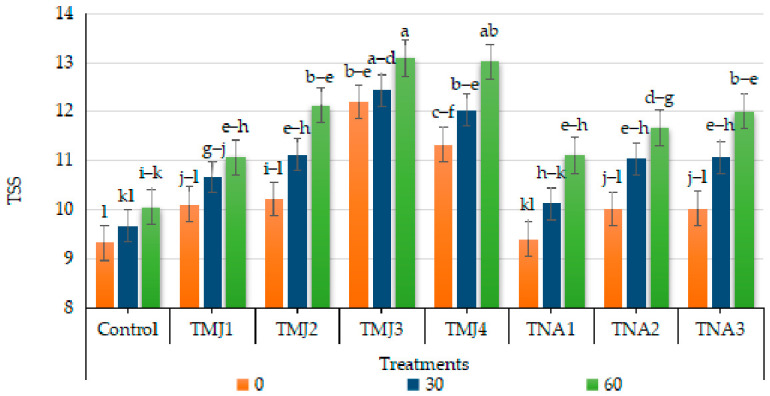
Effect of sonicated nanoemulsion integrated with methyl jasmonate and 1-Naphthalein Acetic Acid on °Brix. The values are presented as mean ± SD. Different letters show a significant difference among treatments at *p* ≤ 0.050. Control: untreated, TMJ1: 2.5 µM L^−1^, TMJ2: 5.0 µM L^−1^, TMJ3: 7.5 µM L^−1^, TMJ4: 10.0 µM L^−1^, TNA1: 300 µM, TNA2: 400 µM, and TNA3: 500 µM.

The total soluble solids (TSS) were highly affected by treatment, days and the interaction between treatment and days (*p* < 0.001). The HSD test showed that TMJ3 and TMJ4 belonged to the highest group (A) with much higher TSS than the control (Group D). High data accuracy is proved by the low CV (2.41%). TSS was gradually increased over time among all treatments; however, the minimum increase (7.18%) was observed until 60 days in the untreated (control) fruits (Figure 6). Total soluble solids (TSS) in preharvest spray treatment, i.e., TMJ2 (5 µM/L), exhibited the most pronounced overall increase (20%) by the end of 60 days, which was a 12.82% higher increase than the untreated (control) fruits. The sonicated nanoemulsions containing 1-naphthalene acetic acid were also effective in enhancing TSS relative to the control. A considerable increase in TSS (19.74%) was recorded in TNA3. Overall, TMJ2 and TNA3 showed superior performance, which suggests the incorporation of plant growth regulators using sonicated emulsion-based delivery to improve fruit juice quality.

#### 3.5.3. Effect of Treatments on Titratable Acidity (TA)

**Figure 7:**
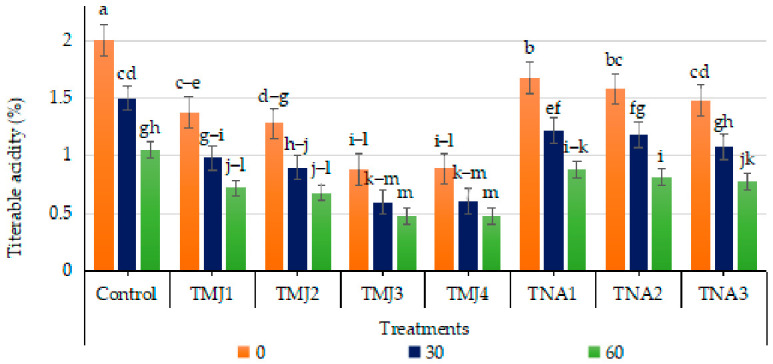
Effect of sonicated nanoemulsion integrated with methyl jasmonate and 1-Naphthalein Acetic Acid on titratable acidity (%). The values are presented as mean ± SD. Different letters show a significant difference among treatments at *p* ≤ 0.050. Control: untreated, TMJ1: 2.5 µM L^−1^, TMJ2: 5.0 µM L^−1^, TMJ3: 7.5 µM L^−1^, TMJ4: 10.0 µM L^−1^, TNA1: 300 µM, TNA2: 400 µM, and TNA3: 500 µM.

Treatment, days, and their interaction had a significant influence on titratable acidity (*p* < 0.001). The HSD test by Tukey showed that the acidity of TMJ3 and TMJ4 (Group G) was the lowest, significantly different compared to the control (Group A). The low CV (2.70) implies accurate measurements. TA gradually declined over time among all the treatments, including the control, reaching the lowest levels during later stages of fruit development, which might be attributed to natural decreases in acidity of fruits during maturation and respiratory utilization of organic acids (Figure 7). The lowest acidity among methyl jasmonate treatments was observed in fruits having treatment TMJ4 (10.0 µM/L), where acidity was recorded as 0.89% at 0 day, which further decreased to 0.61% after 30 days, reaching 0.48% after 60 days, representing the minimum value among all treatments. Similarly, 1-naphthalene acetic acid treatments also accelerated the reduction in TA compared to the control treatment. The maximum decrease in TA was observed in the treatment TNA3 (500 µM), reaching until 0.78% after 60 days, whilst the control treatment exhibited 1.05% after 60 days. These results reflected the efficacy of sonicated nanoemulsions containing methyl jasmonate and 1-naphthalene acetic acid in controlling acid metabolism in the fruits.

#### 3.5.4. Effect of Treatments on Sugar–Acid Ratio

**Figure 8:**
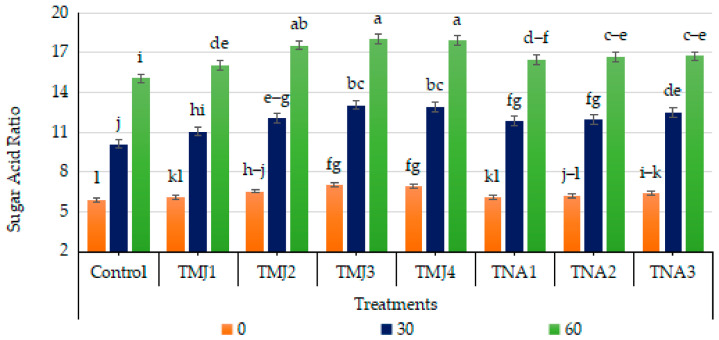
Effects of sonicated nanoemulsion integrated with methyl jasmonate and 1-Naphthalein Acetic Acid on the sugar–acid ratio. The values are presented as mean ± SD. Different letters show a significant difference among treatments at *p* ≤ 0.050. Control: untreated, TMJ1: 2.5 µM L^−1^, TMJ2: 5.0 µM L^−1^, TMJ3: 7.5 µM L^−1^, TMJ4: 10.0 µM L^−1^, TNA1: 300 µM, TNA2: 400 µM, and TNA3: 500 µM.

Treatment, days and their interaction exhibited a highly significant impact on the sugar–acid ratio (*p* < 0.001). The Tukey HSD test indicated that TMJ3 had the highest ratio (Group A), then TMJ4, with the control having significantly lower values. The very small CV (0.38%) points to a high level of smoothness in the experiment. Sonicated nanoemulsions containing methyl jasmonate and 1-naphthalene acetic acid significantly improved the balance of fruit flavor through the concerted effect of sugar deposition and organic acid depletion (Figure 8). The sugar–acid ratio increased during fruit development in control treatments; however, the values remained lower compared to treated fruits. The highest sugar–acid ratio among methyl jasmonate treatments was observed in fruits treated with TMJ4 (10.0 µM/L), where the sugar–acid ratio was recorded as 6.92 at 0 days, which further increased to 12.92 after 30 days, and reached up to 17.92 after 60 days, depicting faster maturation of fruits and biochemical transformation. The sugar–acid ratio was also improved significantly after treatments of fruits with 1-naphthalene acetic acid, i.e., TNA3, showing the maximum increase of 16.72 after 60 days. The pronounced rise in the sugar–acid ratio in both growth regulator treatments proved the efficiency of sonicated emulsion-based delivery in enhancing the taste profile and hence marketability of fruits.

#### 3.5.5. Effect of Treatments on Total Phenolic Contents

**Figure 9:**
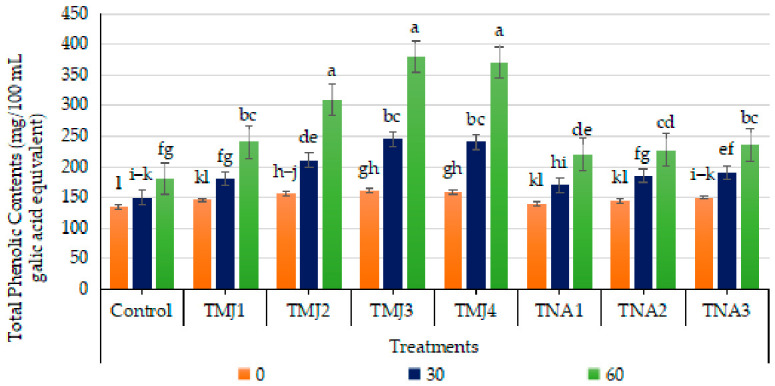
Effect of sonicated nanoemulsion integrated with methyl jasmonate and 1-Naphthalein Acetic Acid on total phenolic contents (mg/100 mL gallic acid equivalent). The values are presented as mean ± SD. Different letters show a significant difference among treatments at *p* ≤ 0.050. Control: untreated, TMJ1: 2.5 µM L^−1^, TMJ2: 5.0 µM L^−1^, TMJ3: 7.5 µM L^−1^, TMJ4: 10.0 µM L^−1^, TNA1: 300 µM, TNA2: 400 µM, and TNA3: 500 µM.

Treatment, days and interaction of the two had a significant effect on total phenolic content (*p* < 0.001). The HSD test revealed that TMJ3 had the largest TPC (Group A), then TMJ4, and then TMJ2, which were significantly greater than the control (Group H). The CV (0.93%) is also low, which means that it is very reliable. Substantial improvement in total phenolic content (TPC) was observed across all treatments after 60 days of spray treatment, while untreated control samples exhibited the least improvement (33.58%). Fruits treated with MeJA treatments indicated more TPC, and in particular, fruits having TMJ3 (7.5 µM/L) treatment displayed 136.23% increased contents (Figure 9). The treatments with 1-naphthalene acetic acid also showed increased TPC concentrations, but at a relatively lower degree as compared to methyl jasmonate treatments. Fruits treated with 1-NAA (TNA3) retained significantly more TPC (235.87 mg GAE/100 mL after 60 days), maintaining 23.32% higher TPC compared to the control. The results indicated the increased efficiency of the treatments using sonicated emulsion-based delivery of methyl jasmonate and 1-NAA.

#### 3.5.6. Effect of Treatments on Total Flavonoid Content (TFC)

**Figure 10:**
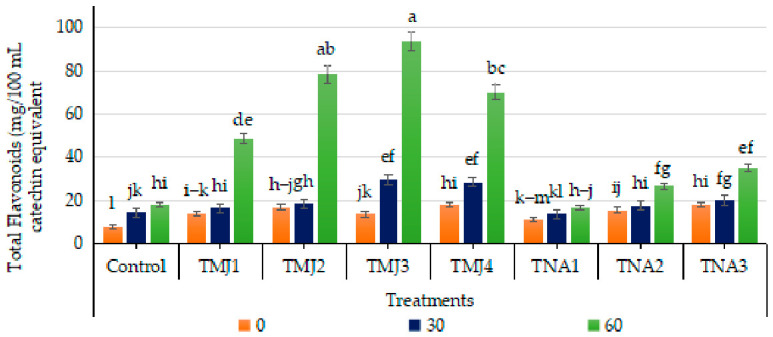
Effect of sonicated nanoemulsion integrated with methyl jasmonate and 1-Naphthalein Acetic Acid on total flavonoids (mg/100 mL catechin equivalent). The values are presented as mean ± SD. Different letters show a significant difference among treatments at *p* ≤ 0.050. Control: untreated, TMJ1: 2.5 µM L^−1^, TMJ2: 5.0 µM L^−1^, TMJ3: 7.5 µM L^−1^, TMJ4: 10.0 µM L^−1^, TNA1: 300 µM, TNA2: 400 µM, and TNA3: 500 µM.

The results revealed that TFC in the juice was consistently increased during fruit development among all the treatments (Figure 10). The treatment, days, and interaction were highly significant (*p* < 0.001). Tucky HSD test grouped TMJ3 on the top (Group A), followed by TMJ4 and TMJ2. The CV (1.31) is very consistent. It was observed that TFC in untreated (control) fruits was 8.12 mg catechin equivalent (CE)/100 mL at the start of the study, which increased to 18.08 mg CE/100 mL after 60 days. Although TFC increased gradually during fruit development in the control group, the values remained lower compared to treated fruit samples. Significant improvement in TFC was observed in TMJ3 treatment, having the highest concentrations (93.75 mg CE/100 mL). Similarly, a significant improvement in TFC (35.25 mg CE/100 mL) was observed in 1-naphthalene acetic acid treatments, i.e., TNA3, after 60 days. Comparison of treatment performance shows that the most potent formulation in increasing flavonoid concentration was TMJ3 and TNA3, which proved the higher potential of methyl jasmonate nanoemulsion in enhancing the TFC in fruits.

#### 3.5.7. Effect of Treatments on Total Flavonols (TFl)

**Figure 11:**
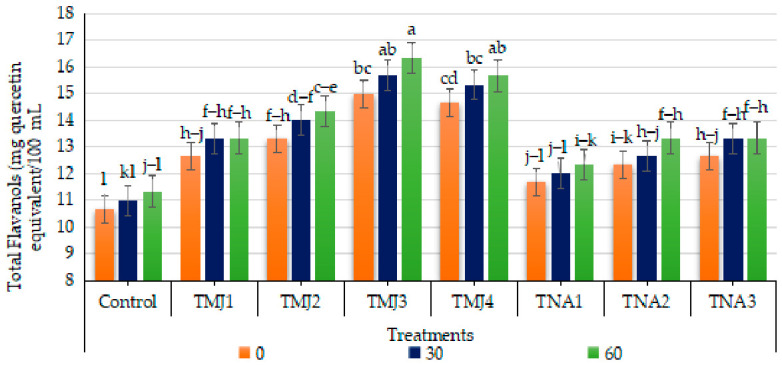
Effect of sonicated nanoemulsion integrated with methyl jasmonate and 1-Naphthalein Acetic Acid on total flavonols (mg quercetin equivalent (QE)/100 mL). The values are presented as mean ± SD. Different letters show a significant difference among treatments at *p* ≤ 0.050. Control: untreated, TMJ1: 2.5 µM L^−1^, TMJ2: 5.0 µM L^−1^, TMJ3: 7.5 µM L^−1^, TMJ4: 10.0 µM L^−1^, TNA1: 300 µM, TNA2: 400 µM, and TNA3: 500 µM.

Treatment and days (*p* < 0.001) had a significant effect on total flavonols, whereas the interaction was nonsignificant. The HSD test grouped TMJ3 and TMJ4 in the highest category (A), which means that they performed better than the control (Group E). Total flavonols (TFl) increased gradually during fruit development in both control and treated samples (Figure 11); however, a comparatively slight increase (6.19%) was observed after 60 days in the control group compared to the treated samples. The highest TFl was observed in TMJ4 (10.0 µM/L), where total flavonol contents were recorded as 15.67 mg quercetin equivalent (QE)/100 mL, representing a substantial improvement (38.31%) compared to the control. Similarly, TNA3 treatment was the most effective in improving TFl (17.65%) in comparison to the control treatment after 60 days. Overall, sonicated emulsions containing 10 µM methyl jasmonate and 500 µM 1-naphthalene acetic acid (TMJ4 and TNA3) were the most outperforming, which depicted that both plant growth regulators integrated with sonicated emulsion were effective in enhancing total flavanols in Tango mandarin fruits.

#### 3.5.8. Effect of Treatments on Total Antioxidant Activity

**Figure 12:**
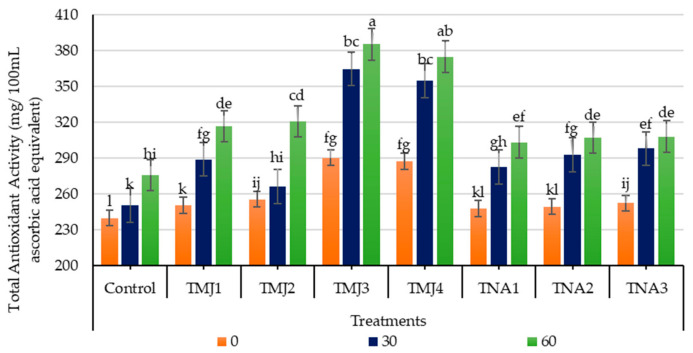
Effect of sonicated nanoemulsion integrated with methyl jasmonate and 1-Naphthalein Acetic Acid on total antioxidant activity (mg/100 g ascorbic acid equivalent). The values are presented as mean ± SD. Different letters show a significant difference among treatments at *p* ≤ 0.050. Control: untreated, TMJ1: 2.5 µM L^−1^, TMJ2: 5.0 µM L^−1^, TMJ3: 7.5 µM L^−1^, TMJ4: 10.0 µM L^−1^, TNA1: 300 µM, TNA2: 400 µM, and TNA3: 500 µM.

Sonicated emulsion treatments of fruits with plant growth regulators considerably enhanced total antioxidant activity during fruit development (Figure 12). Treatment, days, and interaction indicated a highly significant effect on total antioxidant activity (*p* < 0.001). Tukey HSD test showed that TMJ3 was the most effective treatment (Group A), then TMJ4. High data precision is confirmed by the low CV (1.21%). In the control group, total antioxidant activity was recorded as 275.88 mg AAE/100 mL, experiencing 14.88% increase after 60 days, whereas the MeJA-treated samples attained 17.81% more TAA compared to the control. Similarly, 1-naphthalene acetic acid treatments of fruits also improved total antioxidant activity compared to the control treatment; however, such improvement was relatively low compared to methyl jasmonate treatments. The improvement was 22.29% in TNA3, indicating 7.41% more antioxidant activity compared to the control treatment. The findings showed that TMJ3 and TNA3 were more effective in enhancing the antioxidant defense mechanism, which may lead to better postharvest quality maintenance.

#### 3.5.9. Effect of Treatments on DPPH Free Radical Scavenging Activity

**Figure 13:**
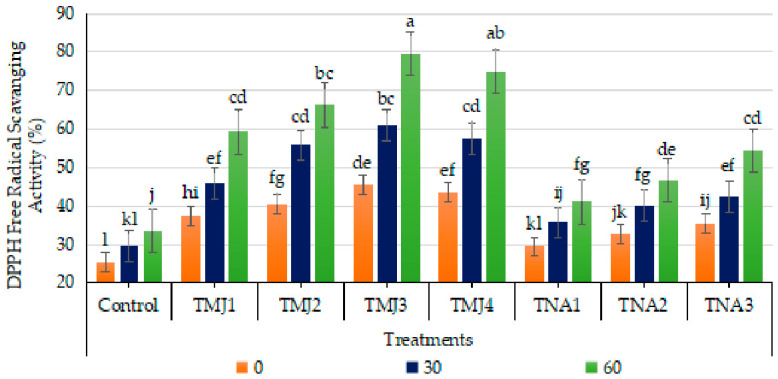
Effect of sonicated nanoemulsion integrated with methyl jasmonate and 1-Naphthalein Acetic Acid on DPPH free radical scavenging activity (%). The values are presented as mean ± SD. Different letters show a significant difference among treatments at *p* ≤ 0.050. Control: untreated, TMJ1: 2.5 µM L^−1^, TMJ2: 5.0 µM L^−1^, TMJ3: 7.5 µM L^−1^, TMJ4: 10.0 µM L^−1^, TNA1: 300 µM, TNA2: 400 µM, and TNA3: 500 µM.

Sonicated nanoemulsion treatments with plant growth regulators, days and their interaction had highly significant effects on DPPH radical scavenging activity (*p* < 0.001). The HSD test showed that TMJ3 was the most efficient (Group A), followed by TMJ4 and TMJ2, which were a lot more active than the control (Group H). The CV (1.99%) indicates reliable experimental data. DPPH radical scavenging activity (RSA) was improved in all treatments of both types of plant growth regulators, i.e., methyl jasmonate and 1-naphthalene acetic acid, throughout the fruit development stages. Among the methyl jasmonate-treated samples, TMJ3 exhibited the maximum increase (75.08%), with a 42.67% increment in DPPH radical scavenging activity (RSA) compared to a 32.41% improvement in the control after 60 days. The 1-naphthalene acetic acid treatment, i.e., TNA3, also showed a significant improvement, preserving 20.80% more DPPH radical scavenging activity (RSA) than the control (Figure 13). The results indicated that methyl jasmonate and 1-naphthalene acetic acid integrated in sonicated nanoemulsions were significantly more effective in improving the DPPH free radical scavenging activity than the control treatment.

## 4. Discussion

The statistical treatment demonstrated that the majority of the detected changes in the parameters of physical, biochemical, and phytochemical conditions were significant (*p* < 0.05) and confirmed the success of the treatments. HSD test further illustrated a distinct separation between groups of treatment, especially MJ3 and TMJ4, which performed better. Most parameters have low values of coefficient of variation (CV%), which means that there is high precision and reliability of the results in the experiments.

### 4.1. Physical Parameters of Fruit Quality

The increase in weight of fruit, its diameter, peel weight, and firmness of treated samples are signs of increased physiological activity and structural growth. The higher fruit size and weight indicated better assimilate allocation and cellular growth that is necessary to promote fruit development [24]. The increased firmness values, especially in TMJ-treated samples, are indicators of enhanced cell wall integrity and slowed softening, and reduced enzymatic degradation of structural polysaccharides. These effects might be related to increased metabolic activity and better control of the processes of ripening. The relatively better performance of methyl jasmonate treatments relative to NAA may indicate a more pronounced effect on stress-related pathways and structural stability, which leads to a longer postharvest quality [25]. Methyl jasmonate enhances cellular proliferation, growth of tissue, and structural and storage metabolites accumulation, which all lead to increased fruit mass. It was also found that the inclusion of methyl jasmonate in the sonicated nanoemulsion probably enhanced the dispersion, cellular penetration, and sustained release of the compound, which increased the uptake efficacy and duration of physiological activity [26]. Methyl jasmonate stimulated carbohydrate-related metabolism and delayed senescence, which led to increased citrus fruit growth [27]. In addition, nano-formulated plant growth regulators enhanced fruit quality and yield by enhancing the efficiency of delivery and the mechanism of controlled release [4]. The increase in fruit weight under the influence of 1-naphthalene acetic acid can be explained by its auxin-like effect, which results in cell elongation, vascular tissue differentiation, and transport of assimilates to growing fruits [28]. In a similar study [29], it was also found that exogenous auxin treatments led to carbohydrate accumulation and increased fruit size in horticultural crops. The effective dosage of synthetic auxins promoted fruit growth, but inappropriate dosages led to reduced growth because of hormonal imbalance [25]. The greater effectiveness of methyl jasmonate in sonicated nanoemulsion improves physiological stimulation, nutrient uptake, and metabolic integration in the tissues of fruits. Plant growth regulators incorporated with nanoemulsion technology have better bioavailability and controlled hormonal activity, which encourages higher fruit growth and quality maintenance.

Methyl jasmonate stimulates cell growth, thickening of parenchymatous tissues, and structural carbohydrate build-up, which results in increased fruit growth. The large fruit diameter at the higher level of methyl jasmonate concentration is a sign of better allocation of assimilates and increased metabolic activity in the immature tissues of the fruit. The addition of methyl jasmonate in sonicated nanoemulsion also enhanced the stability of the compounds, increased absorption rate and release rate, thereby reinforcing its physiological activity and increasing the growth stimulation time. In a similar study [30], it was found that methyl jasmonate was able to boost grapefruit growth by improving antioxidant protection and stress resistance. Asghar et al. [31] observed the growth-promoting effect of pomegranate fruits with the application of methyl jasmonate by regulating carbohydrate metabolism and hormonal balance due to the enhanced delivery and sustained release mechanisms [6]. The increase in fruit diameter with 1-naphthalene acetic acid treatment can be explained by the auxin-mediated cell elongation, vascular differentiation control, and carbohydrate translocation regulation [32]. Auxins promote the transportation of nutrients to the developing fruits and also promote cell growth that enhances the size of the fruit [33]. It was observed that ideal levels of synthetic auxins enhanced fruit production and growth, whereas unsuitable levels led to a decrease in physiological efficiency because of hormonal distress [25]. Thus, findings of the present study validated the synergistic use of methyl jasmonate or 1-naphthalene acetic acid in sonicated nanoemulsion in improving the fruit diameter.

Moreover, it was also reported that methyl jasmonate increased assimilation to epidermal and sub-epidermal strata, which led to an increase in structural reinforcement of tissues of the fruits [34]. Incorporation of methyl jasmonate in sonicated nanoemulsion probably enhanced stability, penetration and controlled release of the compounds, resulting in enhanced physiological efficacy and extended tissue growth response. Li et al. [35] noted that methyl jasmonate increased peel thickness in pitaya fruit because of the induction of antioxidant defense and carbohydrate deposition. It was also reported that nano-formulated plant growth regulators enhanced tissue formation by increasing absorption efficiency and extended delivery of active compounds [36]. The increase in peel weight of fruits after application of 1-naphthalene acetic acid may be explained by the fact that it regulates cell growth and vascular development in peel tissues through auxin. Another study reported that application of auxin led to increased development of the citrus peel, as it increased the cell expansion and redistribution of assimilates [27]. Carbohydrate storage and structural tissue development were enhanced by the use of auxin [37]. The optimal level of synthetic auxin improved the peel properties and fruit development, and the inappropriate level worsened physiological efficiency because of hormonal disproportion [38]. The results of the present investigation highlighted the fact that interactions between plant growth regulators and nanoemulsion enhanced fruit tissue development and fruit quality.

The increase in fruit firmness by means of methyl jasmonate treatments can be attributed to its control of plant defense mechanisms and cell wall metabolism. This increased firmness in the presence of a higher concentration of methyl jasmonate indicates that the cell wall components are reinforced and less pectic substances are broken down during storage. The addition of methyl jasmonate to sonicated nanoemulsion probably increased the dispersion of the compound, cellular absorption, and sustained delivery, producing better physiological efficacy and longer tissue stability. Similar findings were presented by Vasquez-Rojas [39], who observed that methyl jasmonate enhanced the firmness of fruits by lowering the activity of cell wall-degrading enzymes and enhancing antioxidant protection. Methyl jasmonate slowed down the softening by regulating ethylene production and preserving the cell wall structure [40]. Nano-formulated growth regulators enhanced firmness by increasing delivery efficiency and controlled release of bioactive compounds [41]. The enhancement in the fruit firmness by treatments with 1-naphthalene acetic acid is attributable to its auxin-modulated control of cellular organization and production of structural polysaccharides [42]. Auxins stabilize cell wall components, increase carbohydrate metabolism, and slow senescence processes, which help to improve fruit texture [43]. The optimal concentrations of synthetic auxin promoted fruit firmness and shelf life, and improper concentrations inhibited physiological efficiency [44]. The results of the present investigation indicated synergistic action of growth regulators and nanoemulsion technology in enhancing the structural stability of fruits.

### 4.2. Juice Quality Parameters

The improvement in the ratios of total soluble solids and sugar–acid, as well as the drop in titratable acidity, are signs of better fruit maturation and fruit flavors. Such changes are associated with increased carbohydrate metabolism and the production of sugars by breaking down organic acids in the process of ripening. The increase in the sugar–acid ratio of the treated fruit indicated a more appropriate ratio of sweetness to acidity, which is paramount in consumer acceptance [45]. The treatments also favored increased juice production, which implied better water absorption and growth of cells in fruit tissues. In general, these findings indicated that the use of plant growth regulators using a nanoemulsion system has a positive effect on the compositional and sensory properties of the fruit. Methyl jasmonate stimulates carbohydrate metabolism and aids in intracellular retention of water, which facilitates cell expansion and also enhances the accumulation of juice in the cells and tissues of fruits. The addition of methyl jasmonate to sonicated nanoemulsion probably enhanced the stability of the compound, penetration, and controlled release, which enhanced its physiological efficacy and increased the quality of fruits. Methyl jasmonate enhanced the content of blueberry juice by increasing antioxidant protection and water retention capacity [4]. Similarly, methyl jasmonate increased fruit maturation and quality of juice by regulating carbohydrate metabolism and osmotic balance [34], whereas Islam et al. [46] found that nano-formulated plant growth regulators enhanced the fruit quality and juice yield with the help of better efficiency in delivery and prolonged release. The increased percentage of juices during the treatment of 1-naphthalene acetic acid can be explained by the fact that it demonstrates auxin-mediated control of cell growth, vascular development, and water and nutrients can be transported into the developing fruit tissues. In addition, Kaur et al. [18] found that the application of auxin increased the mandarin juice content by accelerating cell expansion and assimilate partitioning. Carbohydrate accumulation and the quality parameters of fruits were improved by the use of plant growth regulators [47]. The gradual improvement in the percentage of juice over time is also indicative of the beneficial role of plant growth regulators in combination with nanoemulsion technology in enhancing the internal quality and stability of fruits.

A study proved that methyl jasmonate accelerates the enzymatic transformation of starch to soluble sugars and antioxidant defense mechanisms, resulting in increased total soluble solid content and fruit sweetness [48]. Methyl jasmonate increased fruit ripening and sugar accumulation by modulating metabolite pathways and osmotic stress [49], whereas Singh et al. [6] found that nano-formulated plant growth regulators increased fruit quality and sugar content by enhancing the delivery efficiency and controlled release mechanisms. 1-naphthalene acetic acid regulates sugar transport and carbohydrate metabolism through its auxin-binding capabilities. Li et al. [26] also observed that the application of auxins positively influenced the soluble solid content by increasing the assimilate partitioning to fruit tissues. Another study also reported that auxin treatments led to better carbohydrate accumulation and other fruit quality parameters in date fruits [50]. The appropriate concentrations of synthetic auxin increased the fruit sweetness and other quality traits [51]. The results of our study also highlighted the efficient use of plant growth regulators via sonicated nanoemulsion technology to enhance the total soluble solids and overall fruit quality.

Methyl jasmonate stimulates the enzyme pathways that take place in the respiratory conversion of organic acids into soluble sugars and other metabolites, and thus lowers acidity and improves flavor. Methyl jasmonate lowered fruit acidity by increasing the activity of antioxidant enzymes and the metabolism of organic acids [52]. Plant growth regulators enhanced fruit quality parameters, such as reduced acidity, by reducing organic acid levels and increasing carbohydrate accumulation [6] using nanoemulsion-based delivery and sustained release systems [53]. The application of 1-naphthalene acetic acid regulates the metabolic processes related to the maturation of fruits and their organic acid metabolism. The use of 1-naphthalene acetic acid enhanced the quality of tomato fruits through the decrease in assimilate partitioning and organic acid metabolism [26]. Similarly, the auxin treatments promoted the accumulation of carbohydrates and the improvement of acidity-related fruit quality parameters in red grapevine berries [54]. The results of the present study highlighted the use of plant growth regulators in sonicated nanoemulsion concerning the reduction in titratable acidity and increase in overall fruit quality.

Research demonstrated that methyl jasmonate activates the enzyme pathways involved in breaking down starch into soluble sugars and also increases the rate at which organic acids break down to enhance flavor balance [35]. The use of plant growth regulators in the form of nano formulations increased fruit quality like sugar–acid ratio [55] due to their efficiency in delivery and release control [53]. Preharvest application of methyl jasmonate improved sugar–acid ratio in Chinese plum-cherry [56], pomegranate [50], Pitaya fruit [35], sweet cherry fruits [52], and raspberries [27]. The increase in sugar–acid ratio in the case of 1-naphthalene acetic acid treatments is due to the fact that it regulates sugar transport, assimilate partitioning, and organic acid metabolism in fruit developing tissues via the auxin pathway [57]. In a study, it was found that application of auxin increased carbohydrate levels and sugar–acid ratio in kiwifruits [58]. Similarly, Asghari et al. [31] observed that preharvest spray of 1-naphthalene acetic acid increased the sugar–acid ratio in Anna apples. Thus, the present findings supported the idea that the use of methyl jasmonate and 1-naphthalene acetic acid in sonicated nanoemulsion may be responsible for enhancing the sugar–acid ratio in fruits.

### 4.3. Phytochemical Parameters

The increased nutritional and functional value of treated fruits is indicated by significant changes in the overall phenolics, flavonoids, flavonols, and antioxidant activity [59]. Hence, these advances indicated that the activation of secondary metabolic processes resulted in enhanced production of bioactive compounds. The increased antioxidant activity also indicated increased oxidative resistance and stress resilience. TMJ3 was the most successful treatment for the best biochemical response. The concerted growth of phytochemicals and antioxidant capacity indicated rigorous improvement of fruit defense systems and nutrition. Methyl jasmonate activates the phenylpropanoid pathway, boosting the production of phenolic compounds that confer antioxidant properties. Liu et al. [60] also found that methyl jasmonate increased phenolic accumulation and antioxidant capacity by stimulating the activity of phenylpropanoid pathway enzymes. Preharvest application of methyl jasmonate also increased the total phenolic content in broccoli [61], pomegranate [62], lemon [63] and Fuji apples [64]. Another study also confirmed that methyl jasmonate increased the nutritional value of fruits by increasing the production of secondary metabolites and the regulation of antioxidant-associated biochemical processes [65] via enhanced nanoemulsion-based delivery efficiency and controlled release properties [6]. 1-Naphthalene acetic acid regulates the metabolic pathways and biosynthesis of secondary metabolites, thereby leading to more accumulation of phenolic compounds [66]. Some more studies also affirmed that treatments of 1-naphthalene acetic acid enhanced the accumulation of phenolic compounds and nutritional quality of fruits [62] like apples [67], mango cv Amrapali [68] and apricot [69]. The results highlighted the efficiency of sonicated emulsion integrated with plant growth regulators in improving total phenolic contents and antioxidant capacity.

Increase in total flavonoid content in fruits by the treatment of methyl jasmonate may be attributed to its regulatory effects in the stimulation of plant defense phenomena and secondary metabolites biosynthesis. It was reported that methyl jasmonate activated the phenylpropanoid pathway, which directly mediates flavonoid formation via triggering the activity of major enzymes like chalcone synthase and phenylalanine ammonia-lyase [70]. Moreover, methyl jasmonate could promote the nutritional value of fruits by increasing the production of secondary metabolites and through the regulation of antioxidant-related biochemical pathways [30]. In some other studies, it was found that methyl jasmonate improved the TFC in blueberry [71], sweet cherry fruits [72], grapefruit [73], Japanese plum [74] and red raspberry [75]. The accumulation of antioxidant compounds was enhanced by the use of 1-naphthalene acetic acid, which stimulated the production of secondary metabolites [76]. The use of 1-naphthalene acetic acid enhanced flavonoid accumulation in fruits [50] as well as the overall nutritional quality of apples [67]. Thus, the findings demonstrated that the integrated use of methyl jasmonate and 1-naphthalene acetic acid by sonicated nanoemulsion is effective in improving TFC in fruits.

Methyl jasmonate activates the phenylprocanoid pathway, thereby increasing the activity of enzymes such as phenylalanine ammonia-lyase and flavonol synthase, which are directly related to the production of flavonols [60]. Similarly, concurrent results were reported in other fruit species like blueberry fruit and red raspberry [71,75]. The increase in the TFl in fruits treated with 1-naphthalene acetic acid might be due to the regulation of metabolic pathways, which are connected to promote the synthesis of flavonols and other antioxidant metabolites [66]. In a similar study, Hussain et al. [50] found that the treatment of 1-naphthalene acetic acid also increased flavonol levels and the nutritional value of fruits. Soethe et al. [77] also observed an increasing trend of total flavanols and antioxidant activity in ‘Baigent’ apples by application of preharvest split application of a plant growth regulator. These results highlighted the synergetic effect between plant growth regulators and nanoemulsion technology in enhancing fruit biochemical composition and postharvest quality.

The increase in total antioxidant activity by the treatments of methyl jasmonate can be linked to its regulatory effect in triggering plant defense mechanisms and biosynthesis of secondary metabolites. In this regard, Asghari et al. [31] observed that methyl jasmonate boosted antioxidant activity by increasing enzyme activity in phenylpropanoid pathways. Another study showed that the use of methyl jasmonate increased the nutritional quality of fruits because secondary metabolite production [26] by emulsion-based delivery efficiency and controlled release characteristics [53]. The accumulation of antioxidant compounds was increased by the application of 1-naphthalene acetic acid [57]. Plant growth regulator treatments enhanced antioxidant capacity and fruit nutritional quality [36], such as apples [67], lemon [52], raspberries [75], and Japanese plum [74]. Thus, the aforementioned findings support the idea that the use of methyl jasmonate and 1-naphthalene acetic acid with the help of sonicated nanoemulsion is an important factor that contributes to the improvement of the total antioxidant activity and quality of fruits.

The increased DPPH scavenging activity of fruits by the treatments of methyl jasmonate might be due to the production of antioxidant metabolites. Methyl jasmonate has been reported to stimulate defense-related gene expression and phenolic and flavonoid biosynthesis, hence enhancing hydrogen-donating capacity and quenching radical activity [58]. In a study, Yuan et al. [78] suggested that 1-naphthalene acetic acid regulated enzyme antioxidant activities and secondary metabolite synthesis, leading to greater redox homeostasis. Moreover, Mwelase et al. [44] demonstrated that synthetic auxin may increase fruit antioxidant capacity during fruit development stages. Sonicated nano-encapsulated elicitors enhanced radical scavenging potential as a result of high bioavailability and controlled release characteristics of bioactive compounds. The progressive growth of DPPH scavenging activity during fruit development stages might be due to the synergistic effect of plant growth regulators and sonicated nanoemulsion technology, which enables sustained release of compounds, their better penetration, improved physiological coordination, and subsequent improved postharvest stability and nutritional quality of fruits [79]. So, the plant growth regulators incorporated into the nanoemulsions have a positive effect on the DPPH free radical scavenging activity.

### 4.4. Nanoemulsion-Based Delivery System and Sonication

The improved efficacy of the treatments can be explained by the delivery system in the nanoemulsion. Sonication results in decreased sizes of the droplets, which leads to a larger surface area and more dispersion stability [80]. This will aid in improved penetration of active compounds into fruit tissues and their controlled and sustained release. The observed changes in physical and biochemical parameters were probably due to the increased bioavailability of the compound methyl jasmonate and NAA [81]. Concerning the application of nanoemulsion-based delivery systems, it has the potential to enhance the efficacy as well as reduce dosages of plant growth regulators. This technology has relevant commercial implications, since it would facilitate more sustainable and focused postharvest management considerations with likely reduced environmental impact.

### 4.5. Implications for Peel Coloration and Degreening

In this study, no direct measurements of peel color parameters were done, but the improvements of the biochemical and antioxidant properties provide indirect evidence of improvements in physiological processes related to peel coloration and degreening. The growth in total phenolics, as well as flavonoid and antioxidant activity, indicated the stimulation of secondary metabolic pathways, which might be strongly correlated with the biosynthesis of pigments and degradation of chlorophyll. Methyl jasmonate is reported to control carotenoid accumulation and chlorophyll degradation pathways underlying the control over change in peel color in citrus fruits [13]. Likewise, synchronized internal and external ripening could be facilitated by modulation of metabolic activity and enhancement of fruit maturation, indicating increased sugar–acid ratios.

Also, the increased physiological activity noted in the treated fruits, such as better firmness and compositional quality, suggests a more controlled ripening process, and the latter process is usually linked to even-colored development. Nanoemulsion-based delivery could have also facilitated these responses by enhancing the uptake and longer action of plant growth regulators [82]. But as the peel color was not directly measured, these interpretations need to be regarded as indicative but not conclusive. It is suggested that future research involving objective color measurements (e.g., colorimeter-measured L, a, b* values) could confirm a direct effect of these treatments on peel coloration and degreening.

### 4.6. Nanoemulsion Characterization

This increased efficiency of the formulation is related to the fact that sonication-assisted emulsification may decrease the size of droplets and enhance the stability of dispersion, resulting in improved contact between bioactive compounds and plant tissues [83]. It should be noted that this study did not directly characterize the formulation concerning its physicochemical nature. In this way, although the preparation technique is comparable to previously employed nanoemulsions, the experimental results of the size distribution of the droplets and the stability values were not measured. This limitation implies that the measured improvements could not be understood in the sense of a well-defined nanoemulsion but in the framework of an emulsion system with improved dispersion characteristics [84]. In this regard, detailed characterization (e.g., dynamic light scattering, zeta potential analysis, and stability measurements) should be used in future studies to verify nanoscale characteristics and further establish the judicativeness of the delivery system. This characterization would also be helpful in commercial and industrial applications.

### 4.7. Limitation of Control Treatment and Interpretation

The current experiment used distilled water as a control treatment. The fruits having control treatment were compared to the fruits having treatments of the nanoemulsion-based delivery system, but the experiment failed to completely differentiate the individual effect of the components of the formulation and the active plant growth regulators [85]. The nanoemulsion system itself, as well as surfactants and stabilizing agents, could also be a factor contributing to the perceived reactions through an increase in surface interaction, permeability, or physiological activity in fruit tissues.

Thus, the enhancements of physical and biochemical, as well as antioxidant properties of the treated samples cannot be attributed solely to either methyl jasmonate or 1-naphthaleneacetic acid, but to the effect of the formulation system and the addition of these two plant growth regulators [86]. This constraint underlines the need to add a control to the formulation without active compounds to isolate the specifics of the carrier system.

Further studies in this area ought to include these controls to adequately distinguish between formulation and compound-specific effects and give a more accurate idea of the mechanisms underlying the improvements observed.

## 5. Conclusions

The results showed that most physical, biochemical, and phytochemical properties of Tango mandarin were greatly (*p* < 0.05) enhanced by the use of plant growth regulators using sonicated nanoemulsion. TMJ3 and TMJ4 had shown the best performance in various parameters as validated by the Tukey HSD test. The fact that the CV values are low for the majority of the parameters also confirms the quality and accuracy of the experimental results. These statistically significant results prove the usefulness of nanoemulsion delivery systems in enhancing the quality of fruit. Future research should focus on scaling up this approach under commercial conditions, evaluating long-term storage performance, and optimizing formulation parameters for broader application across different fruit crops.

## Figures and Tables

**Table 1:** Treatment plan of sonicated nanoemulsion for preharvest spray.

Treatment	Growth Regulator	Concentration	Replications
T0	Control (Water)	—	3
TMJ1	Methyl Jasmonate	2.5 µM L^−1^	3
TMJ2	Methyl Jasmonate	5.0 µM L^−1^	3
TMJ3	Methyl Jasmonate	7.5 µM L^−1^	3
TMJ4	Methyl Jasmonate	10.0 µM L^−1^	3
TNA1	NAA	300 µM	3
TNA2	NAA	400 µM	3
TNA3	NAA	500 µM	3

## Data Availability

The original contributions presented in this study are included in the article. Further inquiries can be directed to the corresponding authors.
